# Facile One-Step Fabrication of Phthalocyanine–Graphene–Bacterial–Cellulose Nanocomposite with Superior Catalytic Performance

**DOI:** 10.3390/nano10091673

**Published:** 2020-08-26

**Authors:** Qiulin Hong, Shiliang Chen

**Affiliations:** Institute of Environmental Sciences, Qianjiang College, Hangzhou Normal University, Hangzhou 310018, China; hongql1201@163.com

**Keywords:** graphene, bacterial cellulose, phthalocyanine, nanocomposite, synergism

## Abstract

It is generally accepted that the convenient fabrication of a metal phthalocyanine-based heterogeneous catalyst with superior catalytic activity is crucial for its application. Herein, a novel and versatile ultrasonic-assisted biosynthesis approach (conducting ultrasonic treatment during biosynthesis process) was tactfully adopted for the direct immobilization of a sulfonated cobalt phthalocyanine (PcS) catalyst onto a graphene–bacterial cellulose (GBC) substrate without any modification. The prepared phthalocyanine–graphene–bacterial–cellulose nanocomposite, PcS@GBC, was characterized by field emission scanning electron microscope (FESEM) and X-ray photoelectron spectroscopy (XPS). The catalytic activity of the PcS@GBC was evaluated based on its catalytic oxidation performance to dye solution, with H_2_O_2_ used as an oxidant. More than a 140% increase of dye removal percentage for the PcS@GBC heterogeneous catalyst was found compared with that of PcS. The unique hierarchical architecture of the GBC substrate and the strong interaction between PcS and graphene, which were verified experimentally by ultraviolet-visible light spectroscopy (UV-vis) and Fourier transform infrared spectroscopy (FT-IR) and theoretically by density functional theory (DFT) calculation, were synergistically responsible for the substantial enhancement of catalytic activity. The accelerated formation of the highly reactive hydroxyl radical (·OH) for PcS@GBC was directly evidenced by the electron paramagnetic resonance (EPR) spin-trapping technique. A possible catalytic oxidation mechanism for the PcS@GBC–H_2_O_2_ system was illustrated. This work provides a new insight into the design and construction of a highly reactive metal phthalocyanine-based catalyst, and the practical application of this functional nanomaterial in the field of environmental purification is also promising.

## 1. Introduction

Metal phthalocyanine complexes (MPcs) are fascinating macrocyclic compounds for many applications [1,2,3], especially in the area of bio-inspired catalysts [4,5,6], considering their structural relations to naturally occurring metal porphyrin complexes. Employing MPcs as versatile catalysts in different types of reactions were extensively studied [7,8,9], and enormous strides were made in this field, while the simple preparation of an MPc catalyst with excellent catalytic performance is still a major challenge.

The catalytic property of MPc is dependent on various factors; to adequately explore its catalytic performance, the fundamental mechanism responsible for the catalytic reaction of MPc should be fully understood. Firstly, MPc has a high tendency to form inactive aggregates, and the immobilization of MPc onto appropriate support is a logical choice to offset this shortage. Secondly, the catalytic process of MPc is crucially dependent on the complexity of electron transfer after coordination between MPc and reactant; thus, providing a microenvironment with outstanding electron transporting property is potentially another strategy to enhance its catalytic activity.

As an important member of carbon allotropes, graphene constitutes a truly two-dimensional planar sheet of sp2-hybridized carbon atoms. This unique structural feature results in outstanding physicochemical properties, including extremely large specific surface area, excellent mechanical property, and high electrical conductivity. The application of graphene in various fields, such as sensors, electrodes, and nanofiller, has been frequently reported [10,11,12,13,14,15]. Particularly, the graphene framework can be employed as an ideal support for the incorporation of various functional materials [16,17,18]. Based on the hierarchical structures of both MPc and graphene, the immobilization of MPc onto graphene is theoretically and experimentally feasible [19,20,21,22,23,24]. In addition, considering that graphene possesses high electrical conductivity and superior electron mobility, synergistically enhanced performance is reasonably expected with the combination of MPc and graphene.

In contrast to individual graphene nanosheets, macroscale graphene-based architectures with three-dimensional structures may be a better choice when used as support for MPc catalyst. The 3D structures can not only improves the dispersion of graphene and reduce the stack of graphene nanosheets, but also promotes the diffusion adsorption of the reactants and improves the accessibility of reactants to the active sites, which is also important to the heterogeneous catalyst [25,26,27].

In our previous work, a graphene-incorporated bacterial cellulose (GBC) nanohybrid was employed as support for the covalent immobilization of tetraamino cobalt phthalocyanine (CoPc) catalyst [28], and an improved catalytic activity of CoPc was found. However, several disadvantages of this technique should be noted. Firstly, MPc was not directly immobilized onto graphene; thus, the communication efficiency between these two electroactive components was significantly reduced, which in return affects the catalytic performance of MPc. Secondly, the covalently binding method is relatively complex, and organic solvent was essential for the immobilization. Moreover, the microstructure of the BC support was inevitably damaged during the chemical treatment.

These issues created the objective of the present work, in which a facile and convenient one-step ultrasonic-assisted biosynthesis approach (conducting ultrasonic treatment during biosynthesis process) [29,30,31] was developed for the direct immobilization of sulfonated cobalt phthalocyanine (PcS) catalyst onto the graphene–bacterial cellulose (GBC) substrate. The prepared nanocomposite, PcS@GBC, was employed as the heterogeneous catalyst for the catalytic oxidation of reactive red X-3B dye molecules. The influence of GBC substrate on the dye removal efficiency of PcS was thoroughly investigated, and the strong interaction between PcS and graphene were identified experimentally by Fourier transform infrared spectroscopy (FT-IR), ultraviolet-visible light spectroscopy (UV-vis), and electron paramagnetic resonance (EPR) technologies and theoretically by density functional theory (DFT) calculation.

## 2. Materials and Methods

### 2.1. Materials and Reagents

Cellulose-forming bacterium *Acetobacer xylinum* (*A. xylinum*) was purchased from BeNa Culture Collection Co. Ltd. (Beijing, China). Graphene solution (0.4–0.5 wt %, with 0.4–0.5 wt % dispersant) was purchased from Aladdin Co. Ltd. (Shanghai, China). Sulfonated cobalt phthalocyanine (PcS, 98 wt %) was purchased from Energy Chemical Co., Ltd. (Shanghai, China) and was purified by a recrystallization process. Reactive red X-3B (RR) was purchased from Shanghai Chemical Reagent Factory (Shanghai, China). 5,5-dimethyl-1-pyrroline-N-oxide (DMPO) was purchased from Sigma Chemical Co. (Saint Louis, MO, USA). All other common chemicals were of analytical grade and purchased from Sinopharm Chemical Reagent Co. Ltd. (Beijing, China).

### 2.2. Preparation of PcS@GBC

PcS@GBC nanocomposite was prepared through direct immobilization of PcS onto the GBC substrate during the biosynthesis process; the typical preparation procedure is as follows. A mixed culture medium composed of 10.0 wt % D-glucose, 1.0 wt % yeast extract, 0.5 wt % peptone, and 1.0 wt % ethanol was sterilized at 121 °C in an autoclave for 30 min. To initiate the biosynthesis process, the bacterium *Acetobacter xylinum* was added into the mixture, and the temperature was kept constant at 30 °C. After cultivation for 24 h, a certain amount of PcS solution and graphene solution were separately added every 24 h, and the resulting mixture was conducted with ultrasonic treatment. After cultivation for 10 days, the sample was collected, incubated in a NaOH solution (0.10 mol/L) for 30 min, thoroughly washed with ultrafiltration water, and subsequently stored in ultrafiltration water for future use. The immobilized PcS amount of PcS@GBC (μmol/g) was calculated as follows:

(1) $$\mathrm{immobilized}\;\mathrm{PcS}\;\left(\mu \mathrm{mol}/g\right)=\frac{n_{1}}{m_{0}}$$

where *n_1_* is the mole number of PcS, which equals the mole number of the Co element and was measured by atomic absorption spectrometry (Thermo Sollar M6); *m_0_* is the weight of the PcS@GBC nanocomposite.

The graphene content of PcS@GBC (mg/g) was calculated as follows:

(2) $$\mathrm{graphene}\;\mathrm{content}\;\left(\%\right)=\frac{m_{2}}{m_{0}}\times 100\%$$

where *m_2_* is the weight of graphene in PcS@GBC, which was measured from the precipitated weight of PcS@GBC after digestion; *m_0_* is the weight of the PcS@GBC nanocomposite.

### 2.3. Characterization

The morphologies and compositions of pure BC, GBC, and PcS@GBC nanocomposites were monitored by field emission scanning electron microscopy (FESEM, Serion, FEI, USA) and X-ray photoelectron spectroscopy (XPS). XPS spectra of all samples were recorded on a Kratos Axis Ultra XPS system with Al (mono) Kα irradiation (hν = 1486.6 eV). The binding energy peaks of all the XPS spectra were calibrated by placing the principal C 1s binding energy peak at 284.6 eV. The functional groups of PcS, graphene, PcS–graphene mixture, and PcS–graphene nanohybrid were characterized by Fourier transform infrared spectra (FT-IR, Brucker Optics, Switzerland). Each spectrum of FT-IR was taken by 32 scans at a nominal resolution of 4 cm^−1^.

The Gaussian09 program package was used to perform the density functional theory (DFT) calculation [32]. The B3LYP-D3 with a 6-31G(d) basis set was used for the geometry optimization.

### 2.4. Catalytic Oxidation Studies and Analysis

To study the catalytic activity of the PcS@GBC nanocomposites, RR dye solution was employed as the model target and H_2_O_2_ was employed as an oxidant. The reaction was carried out in a stirred tank glass reactor and placed in a thermostatic water bath with the temperature set to 50 °C. The typical composition of the reaction mixture was 5 mL of RR dye solution (initial concentration 100 μmol/L) and 0.75 mg of PcS@GBC nanocomposite (immobilized PcS: 43 μmol/g, graphene content: 20.50%). The pH value of the RR solution was adjusted to the desired value by using 1 mol/L HClO_4_ and 1 mol/L NaOH. To initiate the catalytic process, a given volume of H_2_O_2_ was added into the above-mentioned reaction mixture. The concentration of RR solution, which is proportional to its maximum absorbance at 539 nm, was monitored by a UV-Vis absorption spectrometer UV-2450. The dye removal percentage of the solution was expressed as the value of (1−C/C_0_), where C is the instant concentration of RR solution, and C_0_ is the initial concentration of RR solution. The catalytic activity of PcS@G/BC nanocomposite was evaluated by the value of dye removal efficiency, which was calculated as follows:

(3) $$\mathrm{Dye}\;\mathrm{removal}\;\mathrm{efficiency}\;\left(\mu \mathrm{mol}/g\right)=\frac{100\mu \mathrm{mol}/L\times 5\times 10^{-3}L\times (1-\left({C/C}_{0}\right)}{7.5\times 10^{-4}g}$$

where (1 − (C/C_0_)) is the percentage of removed RR dye after treatment. The EPR signals of radical spin-trapped by 5,5-dimethyl-1-pyrroline-N-oxide (DMPO) were detected with a Bruker-A300 X-band EPR spectrometer (Bruker, Karlsruhe, Germany).

To test the stability of PcS@G/BC for cyclic runs, the heterogeneous catalyst was recycled after treatment, thoroughly washed with ultrapure water, and vacuum dried at 25 °C for 24 h for the next use.

## 3. Results and Discussion

### 3.1. Materials Characterization

**Figure 1:**
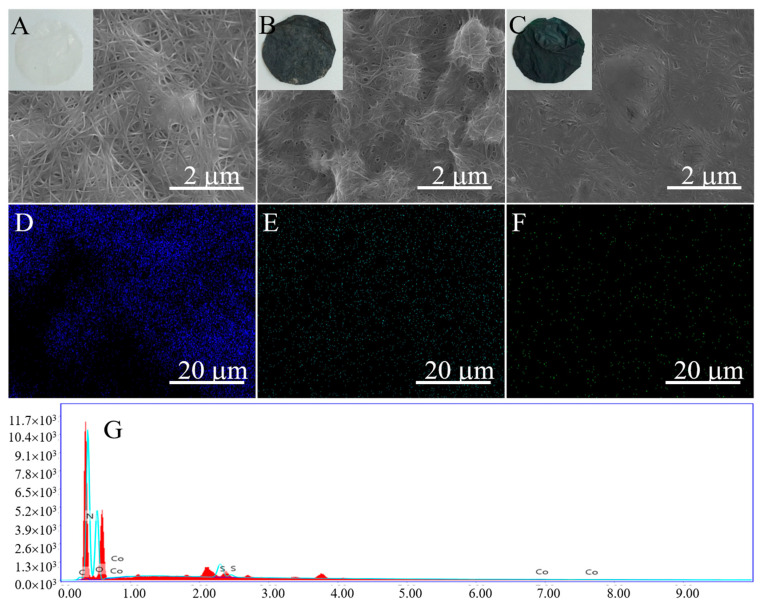
Field emission scanning electron microscope (FESEM) and optical images (inset) of (**A**) bacterial cellulose (BC); (**B**) graphene–bacterial cellulose (GBC), and (**C**) the prepared phthalocyanine–graphene–bacterial–cellulose nanocomposite (PcS@GBC); the elemental mapping images of N, S, and Co elements (**D**–**F**) and EDS spectrum (**G**) of PcS@GBC.

PcS@GBC nanocomposite was prepared by the direct immobilization of PcS onto the GBC substrate. The macro- and microstructures of BC, GBC, and PcS@GBC were observed by digital images and FESEM, respectively. As expected, the pure BC membrane shows a white color (inset of Figure 1A), and an interconnected three-dimensional (3D) network morphology was found (Figure 1A), which was important for the good dispersion of graphene and the subsequent immobilization of PcS. With the incorporation of graphene, the membrane turns to dark black (inset of Figure 1B), and the adsorption of graphene onto BC can be easily observed (Figure 1B). After PcS was immobilized onto GBC, the resulting PcS@GBC membrane displays a deep green color (inset of Figure 1C), and the morphology became much denser compared with that of GBC (Figure 1C). In addition, the elemental mapping images (Figure 1D–F) and the energy dispersive X-ray spectroscopy (EDS) spectrum (Figure 1G) of PcS@GBC clearly show the distribution and existence of N, S, and Co elements, indicating the uniformly immobilization of PcS onto GBC and the successful preparation of the PcS@GBC nanocomposite.

**Figure 2:**
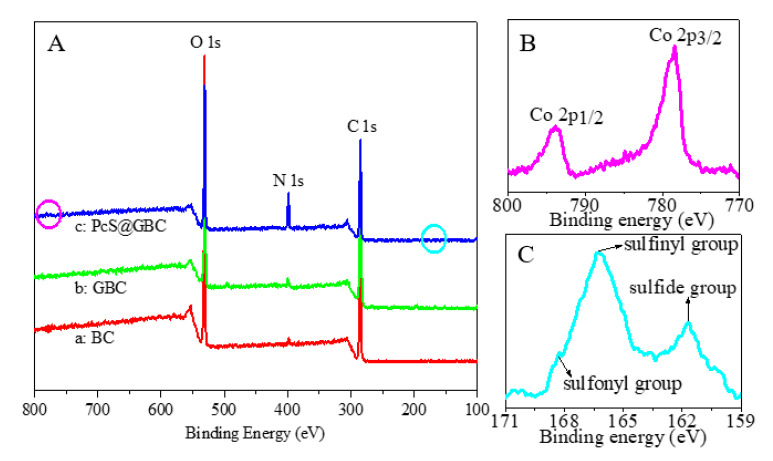
(**A**) XPS spectra of a: BC, b: GBC, and c: PcS@GBC; the details of the Co region (**B**) and S region (**C**) of PcS@GBC.

The chemical compositions of pure BC, the GBC nanohybrid, and the PcS@GBC nanocomposite were monitored by XPS. For BC, the characteristic peaks at 284.6 eV and 531.6 eV were ascribed to the binding energies of C 1s and O 1s, respectively (Figure 2A(a)). When graphene was incorporated into BC, the decrease of O 1s peak intensity (with decreasing the O atomic ratio from 44% to 28%) and the increase of C 1s peak intensity (with increasing the C atomic ratio from 55% to 71%) were found (Figure 2A(b)). For PcS@GBC, the marked increased peak at 398.6 eV was observed, which was the typical signal of N 1s (Figure 2A(c)). A Co 2p_3/2_ peak and Co 2p_1/2_ peak located within the range of 777–781 eV and 792–796 eV were detected, implying the existence of a Co element for PcS@GBC (Figure 2B). In addition, the S2p peaks spinning at 166.4 eV and 161.7 eV correspond to the sulfinyl group and sulfide group, and the binding energy located at 168.2 eV can be ascribed to sulfonyl group of PcS (Figure 2C). The detection of S and Co elements further verified the successful preparation of the PcS@GBC nanocomposite.

### 3.2. Study of Interaction between PcS and Graphene

**Figure 3:**
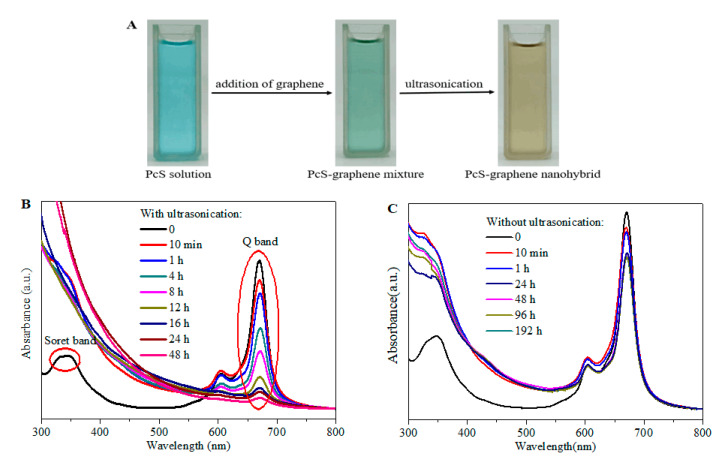
(**A**) Color changes of sulfonated cobalt phthalocyanine (PcS) solution with the addition of graphene and the ultrasonic treatment; (**B**) Effect of ultrasonic time on the UV-vis absorption spectrum of the PcS–graphene nanohybrid; (**C**) Effect of time on the UV-vis absorption spectrum of the PcS–graphene mixture.

The interaction between PcS and graphene is of vital importance to the successful preparation of the PcS@GBC nanocomposite and the subsequent catalytic performance; therefore, the detailed interaction process between these two nanocomponents is urged to be thoroughly understood. Figure 3A shows the digital images of the color changes of the PcS solution after the addition of graphene and subsequently the ultrasonic treatment. The PcS solution exhibited a color of brilliant blue, and the color turned to green when graphene was added. Interestingly, the color of the PcS–graphene mixture has a dramatic change when ultrasonic treatment was carried out; a yellow-colored solution was formed with ultrasonication for 4 h.

UV-vis absorption spectroscopy was employed to further understand the interaction between PcS and graphene (Figure 3B). The PcS solution showed a strong Q-band characteristic peak centered at 670 nm, which was the result of the π–π* transition of mobile electrons of PcS from the ground state to the first excited state (s0→s1) [33,34]. An additional weak vibrational satellite band centered at 605 nm was the result of intermolecular aggregations between the PcS units. The Soret band characteristic peak in the ultraviolet light region was also observed, which can be attributed to the transition from the ground state to the second excited state (s0→s2). When graphene was added, the decreased intensity of the Q-band peak with ultrasonic time was observed, indicating the existence of the strong interaction between PcS and graphene. Furthermore, the red shift of the Q-band of the PcS–graphene nanoconjugate was also noticed (Figure S1), which also indicated the change of the physicochemical property of PcS by graphene and the electronic interaction between the two nanocomponents.

For comparison, when no ultrasound was applied, an approximately 10% decrease of absorption strength of the Q-band of the PcS–graphene mixture was found within 1 h (Figure 3C). While a further increase of time has little effect on the absorption intensity of the mixture, the spectrum keeps almost intact, even prolonging the mixture time to 192 h. In addition, the observation of the shoulder peak centered at 605 nm suggested that some of the PcS molecules remain in the aggregation state. This result implies that the ultrasonic treatment was effective and indispensable for the good dispersion of PcS molecules onto the graphene nanosheet.

**Figure 4:**
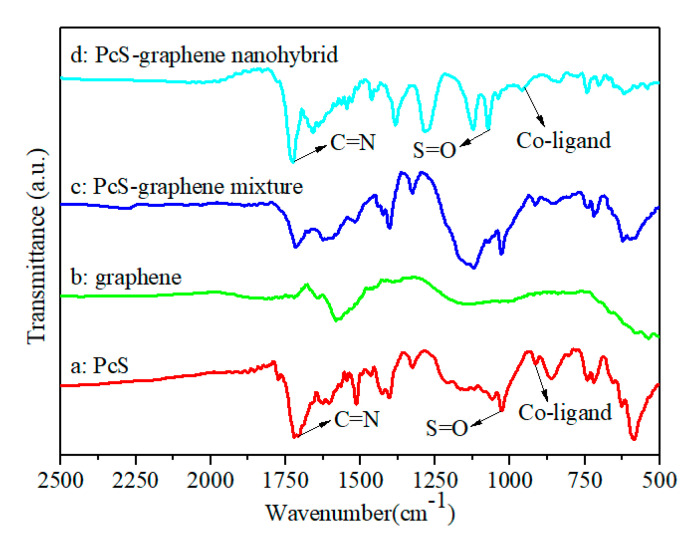
Fourier transform infrared spectroscopy (FT-IR) spectra of (**a**): PcS, (**b**): graphene, (**c**): PcS–graphene mixture, and (**d**): PcS–graphene nanohybrid.

Fourier transform infrared (FT-IR) spectroscopy is an effective technique to characterize the functional groups of samples. Figure 4 shows the FT-IR absorption spectra of PcS, graphene, the PcS–graphene mixture (product without ultrasonication), and the PcS–graphene nanohybrid (product with ultrasonication). For pure PcS (Figure 4a), the characteristic peak located at 915 cm^−1^ was associated with metal–ligand vibration, which revealed the coordination between the 3d unoccupied orbital of Co and the four surrounding nitrogen atoms in the pyrrole rings [34,35,36]. The characteristic peaks at 622cm^−1^, 1326 cm^−1^, and 1514 cm^−1^ represents the skeleton structure vibration of PcS, the C=C stretching vibration of the aromatic nucleus, and the C–C strectching vibration of pyrrole. The characteristic peaks at 1719 cm^−1^ and 1028 cm^−1^ were attributed to the C=N stretching vibration and S=O stretching vibration of PcS, respectively. For graphene, the absorption peaks that appeared at 1580 cm^−1^ and 1210 cm^−1^ were attributed to the skeletal vibration of graphene nanosheets (Figure 4b) [37,38]. All these peaks can be found for the PcS–graphene mixture (Figure 4c). In contrast, an obvious blue shift of the characteristic peaks of C=N (1725 cm^−1^), S=O (1073 cm^−1^), and cobalt–ligand (960 cm^−1^) was found for the PcS–graphene nanohybrid (Figure 4d), which provides further evidence of the strong interaction between PcS and graphene and the formation of a PcS–graphene nanohybrid with the ultrasonic treatment.

**Figure 5:**
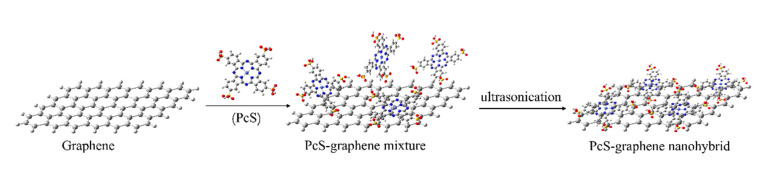
Schematic diagram of the interaction process of PcS and graphene with ultrasonic treatment.

Based on the above-mentioned results of UV-vis and FT-IR spectroscopy measurements, a schematic diagram of the interaction process between PcS and graphene is shown in Figure 5. Without external forces, the PcS molecules were randomly adsorbed onto the surface of graphene, and the interaction between these two nanocomponents was relatively weak. When ultrasonic treatment was conducted on the PcS–graphene mixture, the likelihood of the optimum contact between PcS and graphene was significantly increased. The structural re-arrangement of PcS results in the stronger conjugated electronic interaction between PcS and graphene.

**Figure 6:**
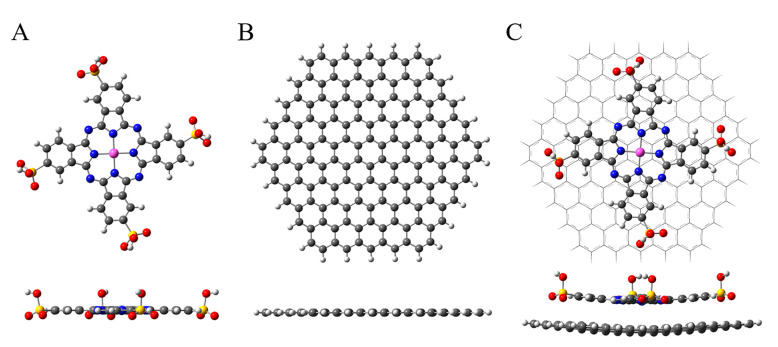
Fully optimized geometries of (**A**) PcS, (**B**) graphene, and (**C**) PcS–graphene nanohybrid. The purple, blue, yellow, red, black, and white balls represent Co, N, S, O, C, and H atoms, respectively.

Density functional theory (DFT) computation, an effective tool to study the electronic structure of nanomaterials, was adopted to fully optimize the structures of PcS, graphene, and the PcS–graphene nanohybrid. As shown in Figure 6A, the geometry of the fully optimized PcS has a planar macrocyclic configuration, and the calculated distance between the Co atom and its surrounding N atoms (dCo–N) was 1.922 Å (Table S1). The geometry of the fully optimized graphene is also planar, as expected (Figure 6B), while the bond distance (dC–C) was calculated to be 1.420 Å, which was consistent with the reference value [39,40]. For the PcS–graphene system, the basal plane adsorption of PcS onto the planar graphene was observed, and the change of the calculated dCo–N for PcS (Table S1) as well as the slight deviation of the geometry of graphene were also noticed (Figure 6C), implying the existence of an interaction between PcS and graphene. The extended, delocalized, and π-conjugated electron system and the unique planar 18 π-electron aromatic structure of the PcS skeleton facilitates this specific interaction.

### 3.3. Catalytic Oxidation Performance of PcS@GBC

**Figure 7:**
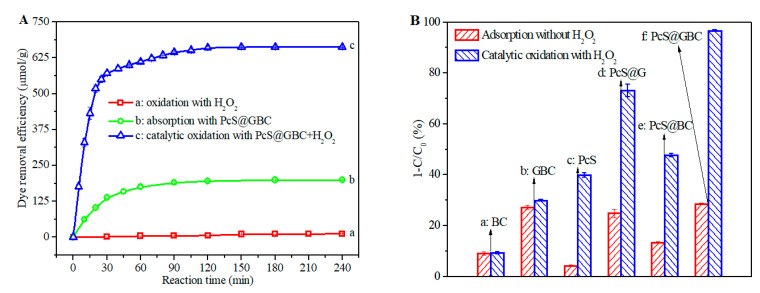
(**A**): Dye removal efficiency of a: H_2_O_2_ (10 mM), b: PcS@GBC (0.75 mg), and c: PcS@GBC (0.75 mg) and H_2_O_2_ (10 mM). (**B**): Comparison chart of dye concentration change by adsorption and catalytic oxidation with a: BC, b: GBC, c: PcS, d: PcS@G, e: PcS@BC, and f: PcS@GBC. Reaction condition: initial dye concentration = 100 μM, pH = 2, T = 50 °C, reaction time = 90 min; the PcS content for c–f was kept the same.

To evaluate its catalytic performance, the prepared PcS@GBC was employed as a heterogeneous catalyst for the decoloration of RR solution, with H_2_O_2_ as an oxidant. No obvious change of dye concentration was found with addition of H_2_O_2_, suggesting that RR dye molecules can hardly be oxidized by H_2_O_2_ alone (Figure 7A(a)). When PcS@GBC was present, a gradual decrease of dye concentration was observed, which can be attributed to the good adsorption capacity of PcS@GBC; the dye removal efficiency was ca. 190 μmol/g with 90 min of adsorption. Meanwhile, a further increase of adsorption time has little effect on the dye removal efficiency, which indicated that the adsorption has reached a dynamic equilibrium (Figure 7A(b)). In contrast, when both PcS@GBC and H_2_O_2_ were present, a sharp decrease of dye concentration was found, and the dye removal efficiency was as high as 600 μmol/g with only 50 min of reaction. The dye removal efficiency reached 660 μmol/g when the reaction time was prolonged to 120 min, i.e., more than 99% of dye molecules were effectively removed (Figure 7A(c)). These results indicated that the PcS@GBC heterogeneous catalyst has excellent catalytic activity, and dye molecules can be efficiently decolorized with the PcS@GBC–H_2_O_2_ reaction system.

To better understand the role of every component of the PcS@GBC (PcS, graphene, and BC) during the catalytic oxidation, the adsorption and catalytic oxidation behavior of different components were briefly studied (Figure 7B). The dye concentration decreased by less than 10% with pure BC, and the same result was found for BC+H_2_O_2_ (Figure 7B(a)), suggesting that BC has a slight adsorption capacity to dye molecules. When GBC was employed, the dye concentration decreased by more than 25%, which can be ascribed to its good affinity to organic dye molecules (Figure 7B(b)). Only 4% of dye molecules can be absorbed by PcS within 90 min, while a ca. 40% decrease of dye concentration can be reached with PcS+H_2_O_2_ (Figure 7B(c)), which shows that the PcS–H_2_O_2_ system is able to catalyze the oxidation of dye molecules. Due to the formation of inactive aggregate in solution, the catalytic activity of PcS was inhibited to some extent. When PcS was immobilized onto graphene, the resulting PcS@G nanohybrid can absorb ca. 25% of dye molecules. With H_2_O_2_ added as an oxidant, the PcS@G–H_2_O_2_ reaction system shows great decoloration capacity: ca. 75% of dye molecules can be catalytic oxidized within 90 min (Figure 7B(d)), which was much higher than that of PcS+H_2_O_2_. The PcS@BC can absorb 13% of dye molecules, and ca. 50% of dye molecules can be catalytic oxidized with the PcS@BC+H_2_O_2_ (Figure 7B(e)), which was higher than PcS+H_2_O_2_ but lower than PcS@G+H_2_O_2_. Interestingly, the catalytic oxidation efficiency of PcS@GBC+H_2_O_2_ was significantly improved when compared with others (Figure 7B(f)). With the presence of H_2_O_2_, more than 95% of dye molecules were catalytically oxidized by PcS@GBC, which was ca. 140% higher than that of the PcS–H_2_O_2_ system. Several reasons were responsible for the substantially improved catalytic activity of PcS. Firstly, the very large surface-to-volume ratio and the distinct 3D nanofibrous network architecture of BC promotes the good dispersion of graphene, which in return improved the immobilization of PcS, and the aggregation of PcS molecules was greatly prevented; thus, the catalytic activity of PcS was accordingly enhanced. Secondly, the electron transfer efficiency plays an important role for the enhancement of catalytic activity of MPc [28,41,42]. Due to its superior electron mobility, the incorporated graphene can facilitate the electron transfer efficiency of the catalytic reaction, which also improved the catalytic activity of PcS@GBC. Therefore, the conclusion is that BC and graphene have a synergistic effect on enhancing the catalytic activity of PcS.

**Figure 8:**
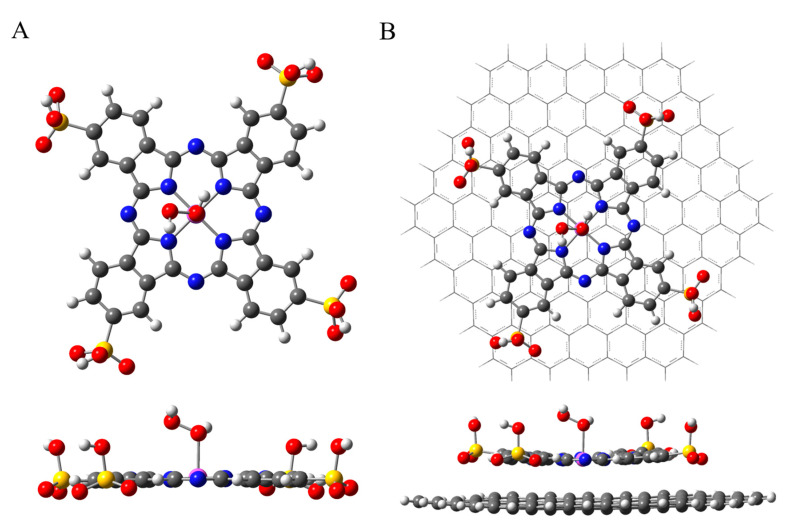
Fully optimized geometries of PcS–H_2_O_2_ in the absence (**A**) and presence (**B**) of graphene. The purple, blue, yellow, red, black, and white balls represent Co, N, S, O, C, and H atoms, respectively.

Figure 8 displays the fully optimized geometries of PcS–H_2_O_2_ in the absence (Figure 8A) and presence of graphene (Figure 8B), respectively. The Co–O distance (dCo–O) between PcS and H_2_O_2_ was calculated to be 2.226 Å (Table S2). For comparison, the dCo–O value was 2.256 Å when graphene was present, which suggests that the existence of graphene obviously influences the electronic interaction between PcS and H_2_O_2_. Furthermore, the O–O bond distance (dO–O) of H_2_O_2_ was calculated to be 1.455 Å when it was chemisorbed onto PcS, and the value was 1.456 Å when graphene was also present; comparing with that of pristine H_2_O_2_ (1.467 Å), the obvious change of dO–O revealed that the H_2_O_2_ oxidant was effectively activated by PcS. Overall, the presence of graphene can change the electronic interaction between PcS and H_2_O_2_, further influencing the catalytic activity of PcS and the resulting catalytic oxidation efficiency of dye molecules.

**Figure 9:**
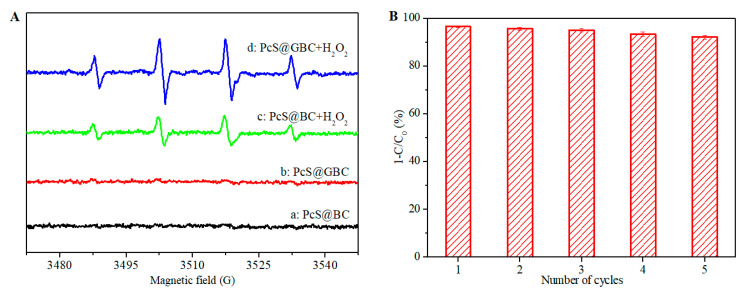
(**A**): EPR spectra of dye solution with existence of a: PcS@BC, b: PcS@GBC, c: PcS@BC–H_2_O_2_, and d: PcS@GBC–H_2_O_2_. (**B**): Cycling experiments of catalytic oxidation of dye solution (initial concentration = 100 μM, pH = 2, T = 50 °C, reaction time = 90 min) with PcS@GBC (0.75 mg) and H_2_O_2_ (10 mM).

To gain insight into the catalytic oxidation mechanism of the PcS@GBC–H_2_O_2_ reaction system, the EPR spin-trapping technique was used to detect the reactive radicals formed during the reaction. As shown in Figure 9A(a), no obvious EPR signal was detected with only PcS@BC. A similar spectrum was observed for the PcS@GBC nanocomposite (Figure 9A(b)), which was in accordance with the analysis of Figure 7, i.e., the decoloration for PcS@GBC alone was purely a physical adsorption process. The characteristic four-line spectrum with a peak intensity of 1:2:2:1 was easily observed for the PcS@BC–H_2_O_2_ reaction system (Figure 9A(c)), which was the well-known spectrum of the DMPO–⋅OH adduct, indicating the formation of a highly reactive hydroxyl radical during the catalytic oxidation. A similar DMPO–⋅OH spectrum can also be found for the PcS@GBC–H_2_O_2_ reaction system (Figure 9A(d)). Moreover, the intensity of the signal was much higher than that of PcS–H_2_O_2_, implying that the GBC substrate is beneficial for the formation of ⋅OH. GBC can not only allow the good dispersion of the PcS catalyst, but also promote the electron transfer efficiency of PcS during the reaction; thus, more ⋅OH can be produced during the reaction, and subsequently, the catalytic activity of PcS was significantly improved.

For practical application, the stability and the recycling performance of the heterogeneous catalyst should be carefully considered. The catalytic activity of the PcS@GBC–H_2_O_2_ reaction system in cyclic utilization was performed, and the result was presented in Figure 9B. The dye removal efficiency has no noticeable decline after five times of repetitive use, and more than 90% of dye molecules can be decolorized within 90 min. These results showed that the PcS@GBC is a promising recyclable catalyst for the consecutive catalytic oxidation of organic pollutants.

**Figure 10:**
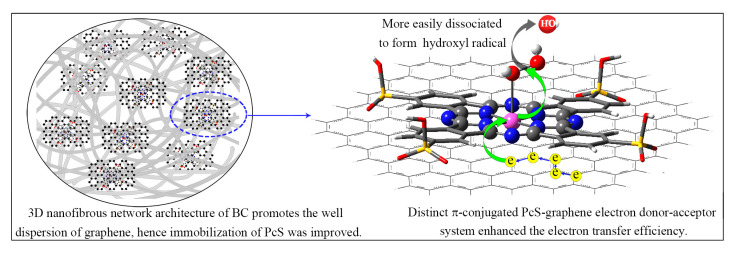
Illustration of synergistic enhancement of catalytic activity of PcS by BC and graphene.

Based on the results presented above, a schematic illustration of synergistic effect of BC and graphene for the enhancement of the catalytic activity of PcS was shown in Figure 10. The super high surface-to-volume ratio and the 3D nanofibrous network architecture of BC guaranteed the good dispersion of graphene, which in return improved the immobilization of PcS molecules and prevented the formation of inactive aggregates. Besides serving as a template to immobilize the PcS catalyst, the distinct π-conjugated PcS–graphene electron donor–acceptor greatly enhanced the electron transfer efficiency of the reaction system. H_2_O_2_, which was chemisorbed on the PcS, was more easily dissociated to form the highly reactive ⋅OH, and therefore, the catalytic activity of PcS@GBC was substantially enhanced.

## 4. Conclusions

In the present study, a green and versatile ultrasonic-assisted biosynthesis approach was presented for a one-step fabrication of highly reactive PcS@GBC heterogeneous catalyst by the direct immobilization of PcS onto the GBC substrate. The interaction between PcS and graphene was verified experimentally by FT-IR and UV-vis spectroscopy and theoretically by DFT calculation. The PcS@GBC heterogeneous catalyst together with H_2_O_2_ can efficiently catalyze the oxidation of dye solution; more than 99% of dye molecules were effectively removed within 120 min. The GBC substrate can promote the dispersion of PcS, facilitate the electron transfer between PcS and H_2_O_2_, and subsequently accelerate the formation of hydroxyl radicals; the dye removal efficiency of PcS@GBC was as high as 660 μmol/g. The results of this work may provide a new standpoint for the design and construction of a heterogeneous MPc-based catalyst, and the practical application of this highly reactive functional nanomaterial in the field of environmental purification was also promising.

## Supplementary Materials

The following are available online at https://www.mdpi.com/2079-4991/10/9/1673/s1, Figure S1: The red shift phenomenon of UV-vis absorption spectrum of the PcS–graphene nanohybrid during the ultrasonication treatment, Table S1: The calculated average distances of C–C for graphene and the average distances of Co–N for PcS, Table S2: The calculated distances of Co–O between PcS and H_2_O_2_ and the O–O bond distance of H_2_O_2_ in the PcS–H_2_O_2_ system and graphene–PcS–H_2_O_2_ system.
