# Supplementary material for: Facile One-Step Fabrication of Phthalocyanine–Graphene–Bacterial–Cellulose Nanocomposite with Superior Catalytic Performance
Source: Nanomaterials (Basel). 2020 Aug 26;10(9):1673. doi: 10.3390/nano10091673 (PMC7558791; doi:10.3390/nano10091673)
Supplement: Supplementary file 1 [file nanomaterials-10-01673-s001.pdf]

nanomaterials

Supplementary Material

Facile One-Step Fabrication of Phthalocyanine–Graphene–Bacterial–Cellulose Nanocomposite with Superior Catalytic Performance

Qiulin Hong and Shiliang Chen *

Institute of Environmental Sciences, Qianjiang College, Hangzhou Normal University, Hangzhou 310018, China; hongql1201@163.com
* Correspondence: bruceblue@zju.edu.cn; Tel.: +86-571-28861372

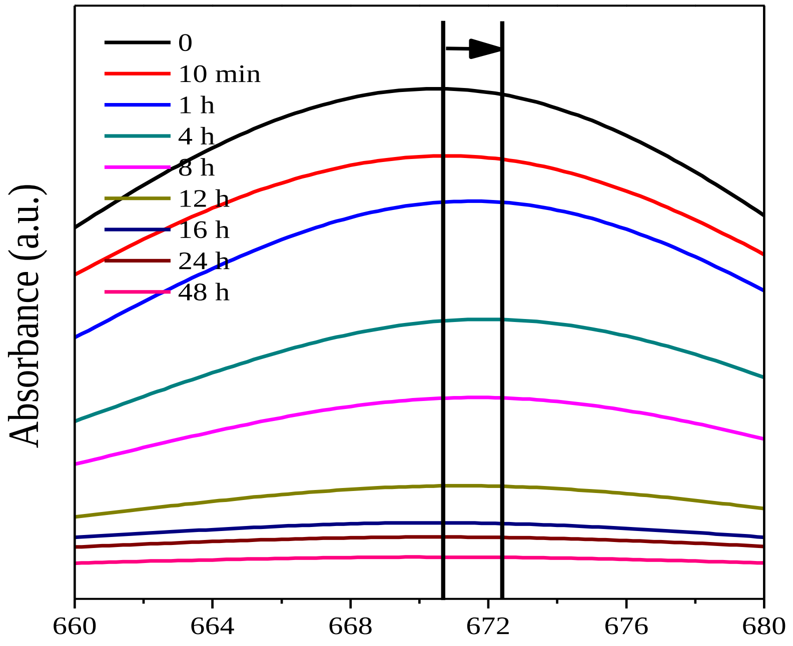

Figure S1. The red shift phenomenon of UV-vis absorption spectrum of PcS-graphene nanohybrid during the ultrasonication treatment.

Table 1. The calculated average distances of C-C for graphene and the average distances of Co-N for PcS.

**System**	**$d_{C\text{-}C}$ (Å)**	**$d_{Co\text{-}N}$ (Å)**
PcS	-	1.92160
graphene	1.41977	-
graphene-PcS	1.41970	1.92577

Table 2. The calculated distances of Co-O between PcS and H_2O_2 and the O-O bond distance of H_2O_2 in the PcS-H_2O_2 system and graphene-PcS-H_2O_2 system.

**System**	**$d_{Co\text{-}O}$ (Å)**	**$d_{O\text{-}O}$ (Å)**
H_2O_2	-	1.46684
PcS-H_2O_2	2.22627	1.45514
graphene-PcS-H_2O_2	2.25568	1.45631
